# Regional Decentralisation in the Greek Health Care System: Rhetoric and Reality

**DOI:** 10.5539/gjhs.v7n6p55

**Published:** 2015-03-30

**Authors:** Athanasios Athanasiadis, Stella Kostopoulou, Anastas Philalithis

**Affiliations:** 1 Health Planning Unit, Department of Social Medicine, Faculty of Medicine, University of Crete, Heraklion, Greece; 2 Department of Economics, Faculty of Economics and Political Science, Aristotle University of Thessaloniki, Thessaloniki, Greece

**Keywords:** decentralisation, Greece, health care reform, regional policy

## Abstract

Decentralisation is a complex, yet basic feature of health care systems in many countries entailing the transfer of authority or dispersal of power in public planning, management and decision making from higher to lower levels of government. This paper describes the attempts made in Greece from 1923 until today to decentralise its highly centralised health care system, drawing on a thorough documentary analysis of legislative acts and official reports regarding regional health policy. The analysis shows that, although decentralisation has been attempted on several occasions, in the end it was abandoned every time. The first ever implementation of a decentralised system of governance in 2001 was also curtailed, resulting in only minor decentralisation of authority and real powers. It is suggested that decentralisation has been impeded by many factors, especially obstruction by opposition from key interest groups, absence of policy continuity between governments, the inability to tackle the bureaucratic and highly centralised system and lack of political will.

## 1. Introduction

Decentralisation as an organisational process has been high on the political agenda in many European health systems over the past decades, denoting a major shift in Europe in the relationship between national, regional, and local control over health sector decision-making. Since World War II, a central thrust of health policy has been to decentralise key dimensions of decision-making authority to increasingly lower levels of government or to Social Health Insurance bodies. In addition, by shifting services to private sector organisations (Saltman, Bankauskaite, & Vrangbaek, 2007), some tax-based systems are also decentralising powers away from the state.

Decentralisation has long been advocated as an efficient process for improving health systems and has been seen as an integral part of broader health reforms aiming to achieve improved equity, efficiency, quality and financial accountability (Bossert, 1998). As a policy and management tool, decentralisation has been very popular in Europe and - to a great extent – it has inspired many attempts for reforms in Greece. The aim of the paper is to identify the attempts to decentralise the Greek health sector and, by use of documentary analysis, to examine the reasons why they have been abandoned. Regarding the current setup, the paper will show how the radical and unprecedented effort to delegate powers to regional structures in 2001 was also curtailed back to the existing, weak co-ordinating competencies that these structures presently hold. The legislation relating to the decentralisation of the Greek health care system will be reviewed from 1923 to today.

## 2. The Different Meanings of Decentralisation

Decentralisation is a complex, multidimensional issue and therefore a difficult research topic to define. Generally, it entails the transfer of authority or dispersal of power in public planning, management and decision making from higher to lower levels of government (Rondinelli, 1981; Collins & Green, 1994; Saltman & Bankauskaite, 2006). Thus, it involves changing the power relationships and distribution of tasks between levels of government (Mills, 1994).

Rondinelli and Shabbir (1983) proposed a four type classification of decentralisation:

- Deconcentration is the redistribution of power to a lower administrative level, i.e. from the central offices, usually located in major metropolitan centres, to peripheral offices of the same public administrative structure (i.e. Ministry of Health)
- Delegation is the transfer of responsibility to a lower organisational level, in the sense that there is a shift of responsibility and authority towards semi-autonomous agencies where boards of directors usually represent distinct corporate interests (labour, business and government).
- Devolution is the transfer of authority to a lower political level, e.g. from the central offices of the Ministry of Health to peripheral administrative structures with their own, usually elected political bodies (e.g. provinces, states and municipalities)
- Privatisation takes place when tasks are transferred from the public domain to the private sector.

In all of the above, although significant authority and responsibility usually remain at the central offices (Rondinelli, 1981; Rondinelli & Shabbir, 1983), they all aim to bring government nearer to the citizens and to encourage community involvement in decision making and/or policy implementation (Mills, 1994).

In the organisation of the health care system, there is often a distinction between functional and geographical (or territorial) decentralisation (Mills, Vaughan, Smith & Tabibzadeh, 1990). Health systems include a number of specific, distinct functions (planning, financing and delivery of services) where each may have different characteristics in terms of the extent of decentralisation that applies (Saltman et al., 2007).

By the end of World War II, most European countries seem to follow parallel paths in decentralising their health care systems. This trend is reflected also in the case of Greece, yet with several differences.

## 3. The Greek Case: Three Phases of the Health System Decentralisation Process

**Figure 1:**
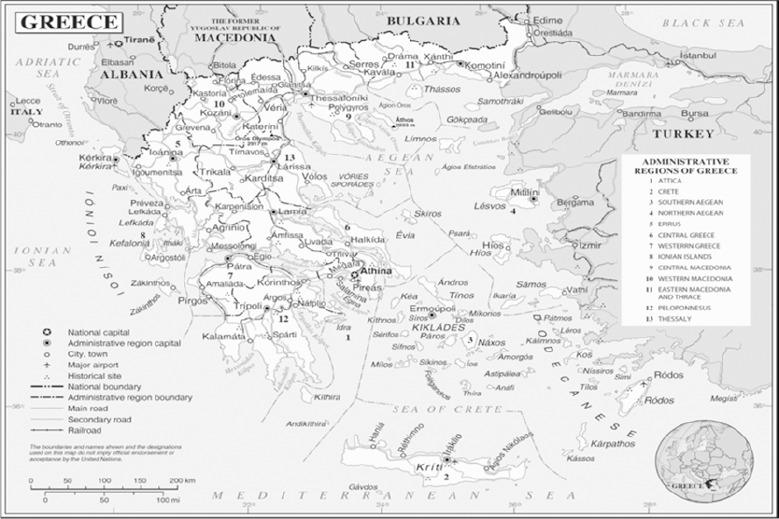
The 13 Administrative Regions (Act 1622/1986 & Presidential Decree 51/87)

Source: United Nations, 2011

Greece, a member-state of the European Union since 1981, is located at the south-eastern end of Europe. It covers an area of 131.957 km^2^ and consists of the mainland and more than 3.000 islands, out of which 169 are inhabited. It has about 15.000 km of coastline and land boundaries with Albania, Bulgaria, FYROM to the north and Turkey to the east. According to the Hellenic Statistical Authority (EL.STAT.), the population of the country in 2011 was approximately 10.8 million. The country is divided into thirteen regions with elected governors (Figure 1), while there are also seven regional administrations, led by a Secretary General who is appointed by the central government (Centre for Health Services Research, 2000; Economou, 2010; Act 3852/2010).

The health system decentralisation attempts in Greece will be reviewed in three phases: from 1923 until 1983; from 1983, when the National Health System was established until 2001; and from 2001 until today.

### 3.1 First Phase- Before the National Health System

#### 3.1.1 The First Attempt in 1923

Following the war in Asia Minor in 1922, Greece had to cope with an influx of 1,221,849 refugees, the vast majority of whom settled down in the two major urban centres of that period, Athens and Thessaloniki. The provision of health and welfare services to this population required a major re-organisation of the state (Pomonis, 1925). Thus, the Ministry of Hygiene and Social Welfare was first established in 1922, and several new state hospitals were created (Niakas, 1993; Moraitis, 2004), while until then different Ministries, municipalities and communities had control over the then few existing public, voluntary, municipal and communal hospitals (World Health Organisation [WHO], 1996).

In this period the first attempt of regionalisation of the health care system was undertaken. In an effort to achieve equity and efficiency in the provision of health services by introducing a certain degree of decentralisation (Pomonis, 1925), the Legislative Decree of 28/10/1923 envisaged the division of the country in five health regions, each having its own regional health board (Charitakis, 1929). This legislation, however, was never implemented. The reasons for abandoning this first attempt are found in the tradition of a strongly centralised government that would be reluctant to cede its powers at a time when the country was facing major economic and social problems and the need to control the allocation of the restricted public funding available for public health infrastructure and services. Provision of financial support and welfare services to the refugees from Asia Minor and the high number of unemployed could not be delegated to lower administrative levels (Zilidis, 1988; Niakas, 1993; Theodorou, Sarris & Soulis, 2001; Moraitis, 2004).

#### 3.1.2 Legislative Decree 2592/1953 and Royal Decree 297/1953

In order to recover from the decade of the devastations of World War II and the Greek Civil war, a large reconstruction process was initiated in the early 1950s that included an emphasis on the reform of the health care system (Philalithis, 1986; Carpenter, 2003). A major step was taken in 1953 with Legislative Decree 2592, designed to create a decentralised health system. This was followed by Royal Decree 297/1953, which defined the spatial regionalisation of the country’s public health system into thirteen health regions, and the decentralisation of its services by establishing regional health boards in every health region (Niakas, 1993) (see Appendix A-Table 1). The regional health boards would express an opinion on health care planning, based on criteria relevant to the local level, such as population demography, morbidity, necessary technical equipment and existing infrastructure. The legislation reflected the perception of a needs-based approach to health care and introduced a certain degree of decentralisation in a system of administration and management that was, until then, highly centralised. The provisions proposed by this legislation were accepted by the Parliament with the support of the majority of the political parties, yet none were ever implemented (Tragakes & Polyzos, 1998; Theodorou et al., 2001).

This is partly due to the fact that, in the early 1950s, Greece was a war-ravaged country that lacked the institutional framework that was necessary for the implementation of the administrative and management changes planned. At the same time, the lack of an economic policy oriented towards a pattern of balanced regional development for the country and, once again, political reluctance to cede any powers to the periphery, constituted major obstacles to the implementation of any significant change (Niakas, 1993). Moreover, the provisions of the legislation were considered to be too radical in the view of the highly conservative mainstream ideology that dominated political life after the end of the Greek Civil war (Theodorou et al., 2001; Moraitis, 2004).

### 3.2 Second Phase- The Initial Stages of the National Health System

Between the late 1950s and early 1960s there were hardly any structural changes in health care (Carpenter, 2003), while the seven-year military dictatorship (1967-1974) is a period characterised by limited investment in the health sector (Centre for Health Services Research, 2000). After the restoration of democracy in 1974 and throughout the 1980s, a new era was beginning with several reforms in the Greek welfare system and the introduction of important legislative acts (Carpenter, 2003).

#### 3.2.1 The Centre of Planning and Economic Research (KEPE) Proposals for Regional Organisation

In 1979, the Centre of Planning and Economic Research (KEPE) issued a landmark report on health services regionalisation that identified the main problems within the health care system. One of the problems of the system that was highlighted was the existence of inequalities between the main urban centres and the rest of the country. The creation of a regionalised system was proposed as a means to reduce inequities in the provision of health services and resources (funding, services and staff), while an analysis of hospital utilisation patterns indicated regional urban centres where major referral hospitals should be built (Centre of Planning and Economic Research [KEPE], 1979).

#### 3.2.2 The Uncompleted Plan ‘Measures for Health Protection’ for a Decentralised Health System

In the same year 1979, a team of experts under the leadership of the then Minister of Health, Spyros Doxiades, constituted a ‘health planning unit’ and prepared a legislative plan entitled ‘Measures for Health Protection’ aiming at the reorganisation of the Greek health care system (Tragakes & Polyzos, 1998; Theodorou et al., 2001; Yfantopoulos, 2003; Tountas, 2008). Among other proposals, the plan proposed the decentralisation of the system into a number of health regions, each administered by a health board responsible for regional health policy planning and for resource allocation within a budget set by the central government (Davaki & Mossialos, 2005).

This plan was the subject of intensive public debate for a period of about three years (1978-1981) and gave rise to strong reactions from medical associations, health workers’ unions and political parties. The Athens Medical Association, which had a conservative leaning leadership, considered it too “revolutionary”, while the Hospital Doctors’ Associations, expressing the views of the centre-left and left opposition political parties considered it too “conservative” (Theodorou et al., 2001). The disagreement between these key interest groups was so strong that the proposed reforms were never even discussed in Parliament (Tragakes & Polyzos, 1998; Davaki & Mossialos, 2005), although the plan was presented to Parliament in 1981, shortly before national elections were due.

#### 3.2.3 Establishment of the National Health System (E.S.Y.)

**Figure 2:**
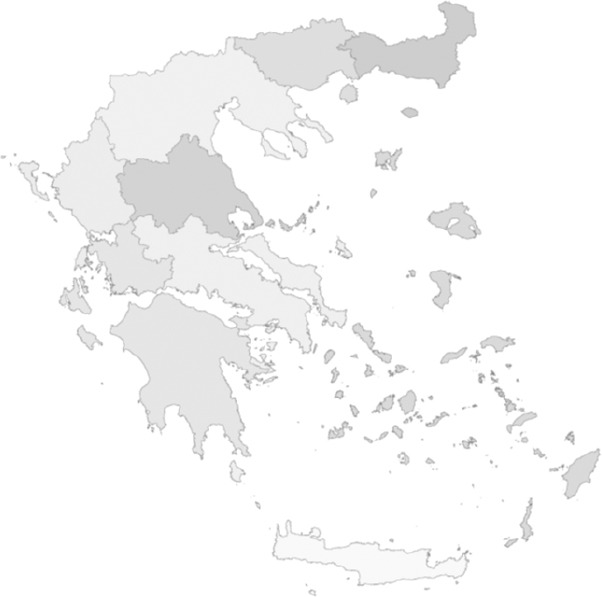
The 9 Health Regions (Presidential Decree 31/1986)

The elections of 1981 brought the socialist party to power, and major legislation for the establishment of the National Health System (E.S.Y.) was approved by Parliament in September 1983 with the enactment of Act 1397. The principal aims of the Act were the provision of universal access to health care services and the achievement of an equitable distribution of health resources; an increase in public health resources and decentralisation of the health system, together with administrative reorganisation were stated as the means to achieve social and geographical equity (Kyriopoulos & Tsalikis 1993). The latter would be accomplished through the division of the country into health regions, with Regional Health Councils that would have an advisory and supervisory authority over health issues in every region, achieving social control through public participation. The Central Health Council (KE.S.Y.) was created as an advisory body to the Minister, charged with the responsibilities of planning health services and coordinating the Regional Health Councils (Kyriopoulos & Tsalikis 1993; WHO, 1996; Theodorou et al., 2001; Tountas, Karnaki & Pavi, 2002; Antonopoulou, 2006). Subsequently, by the Presidential Decree 31/1986, Greece was divided into nine health regions (Figure 2) (see Appendix A-Table 1) (Yfantopoulos, 2003).

However, the creation of the Regional Health Councils was abandoned in early 1987 when the decision was taken not to proceed further with the implementation of the development of the National Health System (Philalithis, 1988). Decision-making and administration management continued to depend upon a centralised and bureaucratic Ministry (WHO, 1996; Tountas et al., 2002; Carpenter, 2003).

#### 3.2.4 Act 2071/1992 and Presidential Decree 370/1992

The elections of 1990 brought the conservative party to power. A new comprehensive law was enacted (Act 2071/1992) that repeated many of the provisions of Act 1397/1983, including the stipulation for decentralisation, albeit with the creation of Regional Health Directorates, in place of the Regional Health Councils of 1983. These Directorates would have a supervising and coordinating function over all the institutions providing health services, as well as an advisory role to the Ministry of Health, regarding the fulfilment of local needs. Once again, a Presidential Decree (370/1992) was issued, dividing the country into thirteen health regions (see Appendix A-Table 1). However, once again, none of the above provisions were implemented as the national elections of 1993 followed shortly after, returning the socialist party to government (Tragakes & Polyzos, 1998).

### 3.3 Third Phase-The Recent Years and the Implementation of Decentralisation

#### 3.3.1 The 17 Regional Health Systems (Pe.S.Y.)

**Figure 3:**
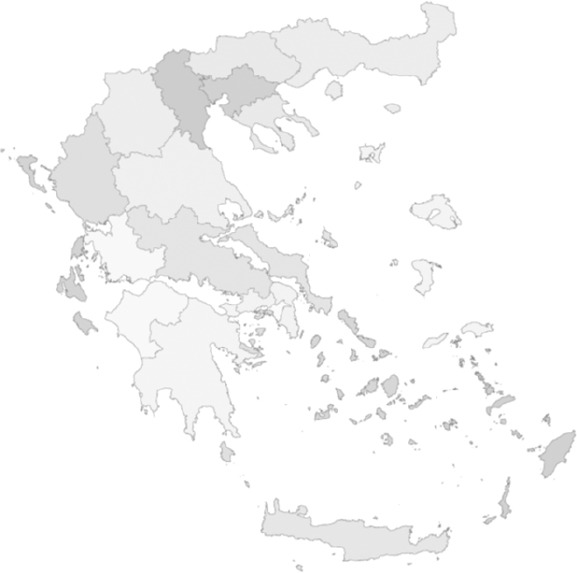
The 17 Health Regions -Pe.S.Y. (Act 2889/2001)

The need for a radical change in the Greek health care system continued to exist. The socialist party, in power since 1993, was re-elected in 2000 and in March 2001 it introduced a new health reform, this time focusing on the regional organisation of the National Health System: Seventeen Regional Health Systems were created (Tountas et al., 2002) (Figure 3) (see Appendix A-Table 1).

These Regional Health Systems were given what were, until that time, unprecedented powers: All National Health System hospitals and primary health care centres in their geographic area of responsibility were incorporated into a single legal body in public law, called Pe.S.Y. They became administratively and economically decentralised units of the single entity, thus granting its board potentially strong powers to influence their functions and services (Tountas et al., 2002; Ballas & Tsoukas, 2004; WHO, 2006). Each Pe.S.Y. had an administrative board, with members named by the Ministry of Health as well as by other stakeholders, chaired by a Chairperson-Director General who was accountable to the Ministry of Health and who was bound by an “efficiency contract” (Liaropoulos, 2001; Aletras, Kontodimopoulos, Zagouldoudis & Niakas, 2007). The Pe.S.Y. were also responsible for developing an operational plan and for setting priorities in order to allocate resources efficiently and to improve efficiency and quality in the delivery of health services at the regional level (Aletras et al., 2007). However, they had no authority regarding major capital investments, paying providers or revising payment methods for hospital staff, all of which remained under the control of the Ministry of Health (Mossialos, Allin, & Davaki, 2005; Aletras et al., 2007). Integrating all health services of the E.S.Y. into one legal entity placed the Directors-General of the health region in a position to plan the health system for the region as a whole, and to by-pass the hospitals which were largely controlled by local hospital managers and health professionals’ trade unions.

The creation of the Pe.S.Y. constituted a radical change in the institutional structure of the health system, since it was meant to be an intermediary between individual hospitals and the Ministry of Health. There was to be a shift in the lines of accountability (and hence a shift of power) away from the Ministry of Health to the regions, while hospitals would be accountable to the Regional Health Administration rather than directly to the Ministry (Mossialos et al., 2005).

On the one hand, many government ministers and national trade unions (particularly those of the hospital doctors and of the civil servants, representing administrative staff in the hospitals) expressed strong disagreement because they feared that their influence on developments in the health sector would be limited. Politicians that formerly used their position to influence individual appointments to hospital boards lost their direct political links with the hospital managers, and had to ask for the intervention of the regional directors, who were directly accountable to the Minister of Health. In addition, financial constraints within the government‘s budget did not permit much latitude to increase support for investing in and expansion of the health sector (Mossialos & Allin, 2005).

On the other hand, hospital doctors and administrative staff in the hospitals themselves opposed the reform for several reasons - mainly because they felt that their direct links with the Ministry of Health were lost (Tountas et al., 2002; Ballas & Tsoukas, 2004; Davaki & Mossialos, 2005). Also, hospital managers felt that the Pe.S.Y. would be monitoring hospital functions and activities too closely, using managerial indicators to evaluate their efficiency, something that the politically appointed hospital boards that previously existed could not do. Finally, the legislation of the Pe.S.Y. contained a number of managerial and administrative inconsistencies (Centre for Health Services Research, 2000; Nikolentzos & Mays, 2008). Opposition to the reform emerged within the government itself and the expected decentralisation of decision-making was never fully implemented (Aletras et al., 2007).

Although the decentralisation effort consisted mainly of devolving political and operational authority to the regional health authorities, it stopped short of shifting full financial responsibility to Pe.S.Y. to the extent that they were not given their own budgets to manage and the Ministry itself still had to validate all financial transactions (Zilidis, 2005; WHO, 2006).

#### 3.3.2 The 17 Regional Health and Welfare Systems (Pe.S.Y.P.)

The situation changed again in 2003 with Act 3106. The Pe.S.Y. were renamed as Regional Health and Welfare Systems (Pe.S.Y.P.) and, in spite of the aforementioned reservations, their responsibilities were extended to include all welfare services within their geographical boundaries that were accountable to the (Deputy) Ministry of Health (Moraitis, 2004; WHO, 2006; Tountas, 2008) (see Appendix A-Table 1). However, the appointment of a new Minister of Health during the next government reshuffle led to the substitution of a large number of Pe.S.Y.P. chairpersons. As a consequence, inconsistencies in the continuation of policy emerged, leading to delays or even cancellations of specific local policies and measures, which weakened the overall decentralisation effort.

#### 3.3.3 The 17 Regional Health Directorates (D.Y.Pe.)

The national elections of 2004 gave rise to a new political scene, since the conservative party returned to power. With Act 3329, which was voted by Parliament in April 2005, the Pe.S.Y.P. were abolished and were replaced by the Regional Health Directorates (D.Y.Pe.) (Moraitis, 2004; Tountas, 2008). The division of the country into seventeen health regions remained the same (see Appendix A-Table 1). Each D.Y.Pe. was a public independent administrative health region managed by a director and a seven-member health board appointed by the Minister of Health. However, the separate legal entities of public law of each hospital (and welfare service) within the region was re-instituted, meaning that hospitals no longer functioned as administratively and economically decentralised units incorporated into the Pe.S.Y.P. Instead the D.Y.Pe. retained only a coordinating function regarding hospital services and development. It was judged that the anticipated control of health expenditure and the expected efficiency through the radical regionalisation scheme of the Pe.S.Y.P. had not been achieved and a body with more limited competences was required (Antonopoulou, 2006; Yfantopoulos, 2007).

Once more, the chairpersons/directors-general were replaced and there was lack of communication and cooperation among the previous and the newly appointed directors that added a further obstacle to the successful implementation of any decentralisation policy.

#### 3.3.4 From 17 Health Directorates (D.Y.Pe.) into 7 Health Regions (Y.Pe.)

In February 2007 a government reshuffle brought a new person to the position of Minister of Health and new legislation (Act 3527/2007) was voted through. This time, there was a fundamental change in the map of health regions: The seventeen Regional Health Directorates (D.Y.Pe.) were merged into seven Health Regions (Y.Pe.), and retained even more limited competences than before, the stated objective being to reduce administrative costs, to initiate greater control and transparency over the budget of public hospitals (Yfantopoulos, 2007) (see Appendix A-Table 1) and to achieve economies of scale through greater efficiency and effectiveness (Economou, 2010). However, the new Health Regions (Y.Pe.) had only limited and rather vague coordinating, supervisory and advisory functions over the hospitals. Another perplexing element of this particular reform was that, although the initially declared intention of the new Minister of Health was to fully abolish the Regional Health Directorates, thus giving an end to the series of legislative efforts that started in 2001, the regional structures were retained without further justification, albeit on a reduced scale (Tountas, 2008). The Administrative Board of the regional authority was abolished and all its powers were vested to the Director of the Health Region and his/her two Deputies (Act 3527/2007). The essential control of the health care system remained with the Ministry (Economou, 2010).

The elections of 2009 led to a change of ruling party, giving the socialist party a clear majority in Parliament. This time, the Y.Pe. were retained without any change in their terms of reference, but the persons holding the positions of Director General and deputy Director General were replaced in accordance with the traditional practices and party political allegiances. The legislative Act of 4052/2012 retained the number of the seven health regions, but changed their geographical boundaries, realigning them in accordance with the boundaries of the seven Regional Administrations.

**Figure 4:**
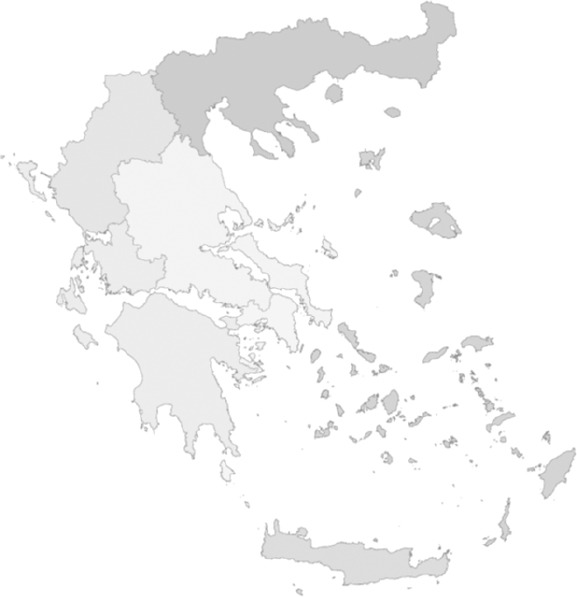
The 7 Health Regions –Y.Pe. (Act 4052/2012)

In a parallel development, changes in the Social Health Insurance funds were implemented. The Act 3918 of 2011 merged the four main social health insurance funds into one organisation, called E.O.P.Y.Y. (National Organisation for Health Care Services Provision), and subsequently almost all other (smaller) social health insurance funds were incorporated into it. As a consequence, the polyclinics of I.K.A. (I.K.A. was the largest Social Security Organisation in Greece before E.O.P.Y.Y.), i.e. its primary health care services of I.K.A., came under the umbrella of the agency charged with the purchase of health services. This was considered as a non-viable situation and in 2014 these services were transferred to the authority of the seven Health Regions (Figure 4) (see Appendix A-Table 1). The Y.Pe. thus became the sole governmental, public provider of health services at a regional level, acting in parallel to the private sector.

## 4. Discussion-Concluding Remarks

In many European countries health decentralisation seems to be the preferred management strategy although the rationale for choosing a decentralised model along with its practical implications may vary across countries and regions (Saltman et al., 2007). The Greek National Health System has traditionally suffered from gross mismanagement and over-centralisation in political, financial and operational terms (Ballas & Tsoukas, 2004). The aim of this paper was to give a brief review of the efforts to achieve health sector decentralisation within Greece, starting within the early 1920’s and continuing up to the present.

The regional decentralisation process in Greece was divided into 3 phases:

1) The early reform proposals, including those put forward during the post-war decades, could be characterised as an ongoing process of trying to reform the system while simultaneously imposing obstacles to implementing these same reforms (Tragakes & Polyzos, 1998). Thus, most reforms remained unfulfilled because of considerable fiscal problems and budget discrepancies. As a result, during this period the Greek health care system underwent gradual changes but not radical reforms.
2) The regionalisation attempts during the years between 1983 and 2001 were characterised by administrative and financial difficulties of the state, strong disagreement by key interest groups and the absence of political consensus on the nature of the reforms (Mossialos & Allin, 2005). Moreover, lack of continuity in health policy among governments of different ideology and planning strategies, even sometimes changes within the same governing party, coupled with a general political unwillingness meant that the two major handicaps of the health system, bureaucracy and centralised decision making, were never confronted.
3) During the decade of 2001-2010, regional structures were finally implemented, but the powers delegated to the Regional Health Systems of 2002 were considered too strong and were curtailed.

In 2010, Greece faced a major fiscal crisis, and had to seek external assistance from the “Troika” of lenders, i.e. the International Monetary Fund, the European Commission and the European Central Bank. In the present context, the cuts to public health spending and cost shifting to patients are relevant (Kentikelenis, Karanikolos, Reeves, McKee & Stuckler, 2014), as well as the requirement set by the “Troika” for further decentralisation, as one of the conditions for the loans that Greece required. Thus, the Act 3852/2010 (the Kallikratis Plan) aimed to transfer responsibility for health care to the new, elected, regional and municipal authorities but, once again, very little has happened in terms of implementing these changes. Instead, Act 4052/2012, already described above, was enacted, which “shuffled” the names and boundaries of the seven Health Regions.

The present paper attempts to explain why the vast majority of these reforms, even when they were approved by parliament, were never put into practice, and on the one occasion when they were implemented, they were never allowed to achieve their full potential. On several occasions, the reforms were short-lived because of subsequent changes of the political party in government or even of the Minister of Health within the same party, thus obstructing the implementation process (Economou, 2010). Political inconsistency, fiscal constraints, insufficient financing and administrative weaknesses posed further significant barriers, hindering and gradually blocking the reforms or resulting in their limited implementation (Tragakes & Polyzos, 1998; Economou, 2010).

Decentralisation entails far-reaching changes that depend on the availability of technical resources in addition to a national institutional and organisational structure on which it can be based. As often underlined in the relevant literature, there cannot be a decentralisation of any kind in health services within a system of public administration which is characterised by a high degree of centralisation (Tragakes & Polyzos, 1998). From all the above it can be assumed that the Greek case is an attempt towards vertical deconcentration referring to the transfer of responsibility and power from a small number to a larger number of administrative actors within a formal administrative structure (Economou, 2010). State-government’s lack of political will to push for regional health policy, a culture of bureaucratically centralised governments, opposition to decentralising reforms from key interest groups and lack of political consensus among political parties in order to provide broader access to health services, have all proven to be the main obstacles to equitable regional distribution of health services and resources. Therefore, the successful implementation of regional decentralisation is still a challenge for the health policy agenda.

We conclude that the most significant problem for Greek health policy is the gap between rhetoric, that is declared objectives and their enactment as law on the one hand, and reality, that is the actual implementation of legislation on the other. The Greek political system seems to stubbornly refuse to accept any degree of effective decentralisation of decision making.

## Legislation

⁃ Legislative Decree 28/10/1923: Organisation of Regional Health Service

⁃ Legislative Decree 2592/1953: Hospital Trusts-Organisation of Medical conception

⁃ Royal Decree 297/1953

⁃ Act 1397/1983: National Health System (E.S.Y.)

⁃ Presidential Decree 31/1986

⁃ Act 1622/1986 : Local Government-Regional Development and Democratic Planning

⁃ Presidential Decree 51/87: Determination of the country’s regions, for the design, planning and coordination of the regional development

⁃ Act 2071/1992: Modernisation and Organisation of the Health System

⁃ Presidential Decree 370/1992: Division of the country into Health Regions

⁃ Act 2889/2001: Improvement and Modernisation of the National Health System and other provisions

⁃ Act 3106/2003: Reorganisation of the National system of Social Welfare and other provisions

⁃ Act 3329/2005 : National system of Health and Social Solidarity and other provisions

⁃ Act 3527/2007: The appointment of legal representatives, supervised by the Ministry of Health and Social Solidarity and other provisions

⁃ Act 3852/2010: The new architecture of the Administration and the Local Government and Regional Administration –Kallikratis Plan

⁃ Act 3918/2011: Structural changes in the Health Care system and other provisions

⁃ Act 4052/2012: Approval of the Draft Financial Assistance Facility Agreements between the European Financial Stability Facility (E.F.S.F.), the Hellenic Republic and the Bank of Greece, approval of the Draft Memorandum of Understanding between the European Commission, the Hellenic Republic and the Bank of Greece and other urgent provisions for the reduction of the public debt and the rescue of the national economy and other provisions

⁃ Act 4238/2014: Primary National Health Network (P.E.D.Y.), change purpose of E.O.P.Y.Y. and other provisions

**Appendix A**

**Table 1:** Health Regions in Greece from 1953-2012

No	PREFECTURE	1953(13)	1986(9)	1992(13)	2001(17)	2003(17)	2005(17)	2007(7)	2012(7)
1	Attiki (A’ & B’ Athina- A’ & B’ Peiraias)	9^th^	1^st^	9^th^	1^st^,2^nd^,3^rd^	1^st^,2^nd^,3^rd^	1^st^,2^nd^,3^rd^	1^st^	1^st^
2	Arta	4^th^	3^rd^	4^th^	9^th^	9^th^	9^th^	6^th^	3^rd^
3	Arkadia	8^th^	2^nd^	10^th^	14^th^	14^th^	14^th^	6^th^	4^th^
4	Argolida	8^th^	2^nd^	10^th^	14^th^	14^th^	14^th^	6^th^	4^th^
5	Achaia	7^th^	2^nd^	7^th^	10^th^	10^th^	10^th^	6^th^	4^th^
6	Aitoloakarnania	7^th^	2^nd^	7^th^	10^th^	10^th^	10^th^	6^th^	4^th^
7	Chalkidiki (Mount Athos)	2^nd^	5^th^	2^nd^	4^th^	4^th^	4^th^	4^th^	7^th^
8	Chania	12^th^	9^th^	13^th^	15^th^	15^th^	15^th^	7^th^	6^th^
9	Chios	10^th^	8^th^	11^th^	8^th^	8^th^	8^th^	2^nd^	5^th^
10	Drama	1^st^	6^th^	1^st^	12^th^	12^th^	12^th^	4^th^	7^th^
11	Dodekanisa	13^th^	8^th^	12^th^	7^th^	7^th^	7^th^	2^nd^	5^th^
12	Evvoia	9^th^	1^st^	8^th^	17^th^	17^th^	17^th^	5^th^	2^nd^
13	Evros	1^st^	7^th^	1^st^	12^th^	12^th^	12^th^	4^th^	7^th^
14	Evrytania	6^th^	1^st^	8^th^	17^th^	17^th^	17^th^	5^th^	2^nd^
15	Florina	3^rd^	5^th^	3^rd^	13^th^	13^th^	13^th^	3^rd^	3^rd^
16	Fokida	6^th^	1^st^	8^th^	17^th^	17^th^	17^th^	5^th^	2^nd^
17	Fthiotida	6^th^	1^st^	8^th^	17^th^	17^th^	17^th^	5^th^	2^nd^
18	Grevena	4^th^	5^th^	3^rd^	13^th^	13^th^	13^th^	3^rd^	3^rd^
19	Ileia	7^th^	2^nd^	7^th^	10^th^	10^th^	10^th^	6^th^	4^th^
20	Imathia	2^nd^	5^th^	2^nd^	5^th^	5^th^	5^th^	3^rd^	7^th^
21	Ioannina	4^th^	3^rd^	4^th^	9^th^	9^th^	9^th^	6^th^	3^rd^
22	Irakleio	12^th^	9^th^	13^th^	15^th^	15^th^	15^th^	7^th^	6^th^
23	Karditsa	5^th^	4^th^	5^th^	16^th^	16^th^	16^th^	5^th^	2^nd^
24	Kavala	1^st^	6^th^	1^st^	12^th^	12^th^	12^th^	4^th^	7^th^
25	Kastoria	3^rd^	5^th^	3^rd^	13^th^	13^th^	13^th^	3^rd^	3^rd^
26	Kefallinia	7^th^	2^nd^	6^th^	11^th^	11^th^	11^th^	6^th^	4^th^
27	Kerkyra	7^th^	3^rd^	6^th^	11^th^	11^th^	11^th^	6^th^	4^th^
28	Kilkis	2^nd^	5^th^	2^nd^	4^th^	4^th^	4^th^	4^th^	7^th^
29	Korinthos	9^th^	2^nd^	10^th^	14^th^	14^th^	14^th^	6^th^	4^th^
30	Kozani	3^rd^	5^th^	3^rd^	13^th^	13^th^	13^th^	3^rd^	3^rd^
31	Kyklades	11^th^	8^th^	12^th^	6^th^	6^th^	6^th^	2^nd^	5^th^
32	Laconia	8^th^	2^nd^	10^th^	14^th^	14^th^	14^th^	6^th^	4^th^
33	Larisa	5^th^	4^th^	5^th^	16^th^	16^th^	16^th^	5^th^	2^nd^
34	Lasithi	12^th^	9^th^	13^th^	15^th^	15^th^	15^th^	7^th^	6^th^
35	Lefkada	7^th^	3^rd^	6^th^	11^th^	11^th^	11^th^	6^th^	4^th^
36	Lesvos	10^th^	8^th^	11^th^	8^th^	8^th^	8^th^	2^nd^	5^th^
37	Magnisia	5^th^	4^th^	5^th^	16^th^	16^th^	16^th^	5^th^	2^nd^
38	Messinia	8^th^	2^nd^	10^th^	14^th^	14^th^	14^th^	6^th^	4^th^
39	Pella	2^nd^	5^th^	2^nd^	5^th^	5^th^	5^th^	3^rd^	7^th^
40	Pieria	2^nd^	5^th^	2^nd^	5^th^	5^th^	5^th^	3^rd^	7^th^
41	Preveza	4^th^	3^rd^	4^th^	9^th^	9^th^	9^th^	6^th^	3^rd^
42	Rethymno	12^th^	9^th^	13^th^	15^th^	15^th^	15^th^	7^th^	6^th^
43	Rodopi	1^st^	7^th^	1^st^	12^th^	12^th^	12^th^	4^th^	7^th^
44	Samos	11^th^	8^th^	11^th^	8^th^	8^th^	8^th^	2^nd^	5^th^
45	Serres	2^nd^	6^th^	2^nd^	4^th^	4^th^	4^th^	4^th^	7^th^
46	Thesprotia	4^th^	3^rd^	4^th^	9^th^	9^th^	9^th^	6^th^	3^rd^
47	Thessaloniki (A’ & B’ Thessaloniki)	2^nd^	5^th^	2^nd^	4^th^, 5^th^	4^th^, 5^th^	4^th^, 5^th^	3^rd^, 4^th^	7^th^
48	Trikala	5^th^	4^th^	5^th^	16^th^	16^th^	16^th^	5^th^	2^nd^
49	Voiotia	9^th^	1^st^	8^th^	17^th^	17^th^	17^th^	5^th^	2^nd^
50	Xanthi	1^st^	7^th^	1^st^	12^th^	12^th^	12^th^	4^th^	7^th^
51	Zakynthos	7^th^	2^nd^	6^th^	11^th^	11^th^	11^th^	6^th^	4^th^

*1953:13 Health Regions;

*1986: 9 Health Regions;

*1992:13 Health Regions;

*2001:17 Health Regions (Pe.S.Y.) [3 Health Regions in Athens, 2 Health Regions in Thessaloniki];

*2003:17 Health Regions (Pe.S.Y.P.) [3 Health Regions in Athens, 2 Health Regions in Thessaloniki];

*2005:17 Health Regions (D.Y.Pe.) [3 Health Regions in Athens, 2 Health Regions in Thessaloniki];

*2007:7 Health Regions (Y.Pe.);

*2012:7 Health Regions (Y.Pe.).
